# American Academy of Dental Science

**Published:** 1892-12

**Authors:** 

**Affiliations:** American Academy of Dental Science


### AMERICAN ACADEMY OF DENTAL SCIENCE.

THE regular monthly meeting of the American Academy of Dental Science was held in the Boston Medical Library Association rooms on June 1, 1892, at 7.30 P.M., President Brackett in the chair.

The paper of the evening was read by Henry S. Chase, M.D., of St. Louis, Missouri; subject, "The Law of Heritage in Regard to the Teeth and Jaws."

(For Dr. Chase's paper, see page 877.)

### DISCUSSION.

*President Brackett.*—The paper of Dr. Chase is before you for discussion. It certainly is a suggestive paper, and makes a broad study of a far-reaching law, and is perhaps quite as profitable in its place as one that considers details of daily practice.

*Dr. Williams.*—Heredity, in my opinion, may be held responsible not only for disproportion between the size of teeth and jaw, but also for strength or weakness of the tooth-substance.

I don't know whether Dr. Chase in his essay strictly meant that "blood did not tell." If he did, I should not agree with him. Heredity of qualities has been almost universally recognized.

*Dr. Kinsman.*—I have an interesting case, showing the development of teeth as affected by the law of heredity.

My wife and I are both without lateral incisors. I have two children,—a girl six years old, who had a perfect set of deciduous teeth, and is now beginning to erupt her second teeth, and a boy, who is minus the right superior deciduous lateral, his superior jaw being a perfect miniature of his father's. I had expected that in the permanent teeth there would be a lateral missing somewhere, but I did not look for its absence in the deciduous teeth. Of course it cannot be possible that he will have a permanent lateral on that side. In my wife's family I do not find anything missing in either of the parents, but every other child is minus the laterals. One aunt is minus the laterals, and retained her superior deciduous cuspids until over fifty years of age. Her superior wisdom teeth were not erupted until after fifty years.

I came across a case recently of a young man of about twenty-five who had four laterals, two of them in the centre of the mouth directly back of the permanent centrals, one of the laterals so placed being considerably larger than either of those that were in position. We frequently meet with cases of one extra lateral, but I never before heard of a case showing two.

*Dr. Williams.*—I received a suggestion from a patient several years ago which, to his mind, seemed to be an explanation of the cause of the lack of the lateral incisors in his son's mouth.

The son was probably twenty-four at the time, and the father asked me if I thought he would ever erupt the laterals. "Probably not; sometimes they are omitted," I said. The gentleman, who had a stock-farm, said that in raising colts the lack of a tooth was thought to be an indication of high breeding. I told him that perhaps that might account for the omission in his son's case.

*Dr. Andrews.*—Some years ago, Dr. Cutter, of New Orleans, wrote an article in which he spoke of a colony of tailless cats which existed somewhere on the Mississippi River. He attributed their peculiar condition to the fact that a certain cat lost her tail, and henceforth bore tailless kittens.

*Dr. Stevens.*—In the State of Maine, where tailless cats exist, they attribute their peculiarity to the fact of their being crossed with rabbits.

*Dr. Williams.*—Contraction of the jaws has been attributed by Dr. O. W. Holmes in part to lack of use, and in part to habits of hasty feeding. On this theory the sturdy Englishman who takes pleasure in chewing his beefsteak is likely to have an ample alveolar arch and a full complement of teeth. But where soft foods are largely used and the teeth are not needed in mastication, there is likely to be a deterioration in the quality of the teeth and lessening of their number.

*Dr. Meriam.*—Dr. Andrews's statement with regard to tailless cats reminds me of one of the curious things which are said about the breeding of rabbits. By crossing two breeds and breeding the offspring of this union to the offspring of two other distinct breeds, it is possible to obtain a rabbit which will hold a distinct strain. With regard to cats, you may have noticed that the ordinary tortoise-shell cat is always a female, and the buff cat is always a male, and they will hold to these colors, no matter how much they are bred.

*Dr. Moffatt.*—One of the most important suggestions made to-night is that data should be collected and kept in the museums of our societies, in order to form a basis for arriving at conclusions as to the subject under discussion. We need models of cases and full notes with reference to them.

*Dr. Smith.*—Irregularities of the deciduous teeth are rare. Dr. Talbot has, in his collection, two or three cases of supernumerary teeth in the deciduous set. I have, in my own collection, a case showing a supernumerary deciduous lateral. I have never read of or seen a case where the deciduous lateral was missing. It seems to be established that irregularities of the teeth are produced by a crossing of types and races. It is stated that in a pure race like the Chinese, Japanese, or North American Indian there are no irregularities. Such deformities are therefore in a sense the result of civilization.

*Dr. Meriam.*—I have in my care a little patient presenting a clear case of protrusion of the lower jaw. It is an irregularity of the deciduous teeth, and I have never seen such a case before. With regard to the matter of heredity, I can call to mind instances where the union of healthy parents has produced feeble offspring. We cannot quite say that the bringing together of certain people will furnish a definite result.

*President Brackett.*—There is much lack of definite views on the subject of heredity. To my mind marked peculiarities are attained through a peculiar heredity and its cultivation. Given a certain line of ancestry, and peculiar characteristics can be developed. The confusion appears in many men's minds that surgical procedures are a matter of considerable consequence. To me they seem of little importance, except as they affect the use of a part. In the State of New Hampshire the farmers have, for many generations, cut off the tails of lambs, but as far as I know there have been no lambs born without tails.

The Jewish custom of circumcision is another case in point. The extraction of teeth has little, if any, influence upon the number of teeth to appear in succeeding generations. But when teeth are lacking through nature's operations, and people having such peculiarities intermarry, then a lack of teeth may be expected in their offspring.

PRESENTATION OF SPECIMENS.

*President Brackett.*—A gentleman, aged about thirty-five, came to me two months ago with these little specimens which I pass around.

They have all the characteristics of calculus. The patient for about two years had been having trouble in the region of the left lower third molar, which tooth had never erupted. On examination several fistulæ were found on the inside of the mouth. There was considerable swelling and a profuse and offensive discharge. After injecting a solution of cocaine and carbolic acid I cut through the soft swollen tissues. A probe disclosed something feeling like a tooth. With this lever-like instrument I was able, without much difficulty, to bring to light this third molar. The crown of the tooth was incrusted with calculus, and hence the origin of the particles first passed around. After the operation, the affected parts returned to a healthy condition in reasonable time.

*Dr. Andrews.*—Dr. Charles H. Osgood, of this city, had a patient several years ago, who was frequently confined to his bed for two or three weeks on account of pain about the face. The best surgeons of Baltimore and Boston had failed to locate the cause of the trouble. Dr. Osgood suspected an unerupted wisdom tooth, and finally succeeded in extracting one which looked very much like the one which Dr. Brackett has shown to-night.

*Dr. Stevens.*—That brings to my mind the case of a young lady who was a patient of Dr. Charles Aspinwall, of Lynn, Mass. She had a sore on her face for which she had been treated by surgeons in vain for a long time. Dr. Aspinwall removed from her a wisdom tooth, extracting it from the outside through the cheek.

WILLIAM H. POTTER, D.M.D.,
*Editor American Academy of Dental Science.*

